# Therapeutic mechanistic framework of mesenchymal stem cell-derived extracellular vesicles in neurodegenerative diseases

**DOI:** 10.20517/evcna.2026.54

**Published:** 2026-09-30

**Authors:** Gunjan Bawne, Magdalena J. Lorenowicz

**Affiliations:** ^1^Department of Advanced In Vitro Model Systems, Biomedical Primate Research Centre, Rijswijk 2288 GJ, The Netherlands.; ^2^Department of Population Health Sciences, Animals in Science and Society, Faculty of Veterinary Medicine, Utrecht University, Utrecht 3584 CM, The Netherlands.

**Keywords:** Neurodegenerative diseases, mesenchymal stromal cells, extracellular vesicles, SARS-CoV-2, signal transduction, neuroinflammation, proteostasis, redox homeostasis

## Abstract

Neurodegenerative diseases are a spectrum of progressive disorders unified by chronic neuroinflammation, oxidative and mitochondrial stress, and accumulation of misfolded proteins that ultimately drive neuronal dysfunction and loss. Despite recurring convergence on these pathogenic processes, therapeutic progress has remained limited, underscoring the need for interventions capable of targeting convergent pathogenic pathways rather than single downstream features. Mesenchymal stromal/stem cells (MSCs) have emerged as promising therapeutic candidates through their paracrine actions that modulate inflammatory and cellular stress responses within the nervous system. Increasing evidence indicates that extracellular vesicles released by MSCs (MSC-EVs) are key mediators of these effects, transferring regulatory RNAs, lipids, and proteins that reprogram recipient cells, attenuate neuroinflammatory cascades, and support neuronal survival. MSC-EVs can recapitulate key therapeutic mechanisms of their parent cells and may offer potential advantages in safety, scalability, and delivery across the blood-brain barrier. While MSC-EVs are promising candidates for treating these diseases, the molecular mechanisms underlying their effects are still emerging. In this review, we synthesise evidence from experimental models to develop a mechanistic framework describing how MSC-EVs modulate interconnected regulatory modules that shape neuroinflammation with blood-brain barrier dysfunction, oxidative and mitochondrial stress, and dysregulated proteostasis with loss of neurotrophic support. We further discuss how this modular perspective may inform strategies to mitigate infection-associated neurodegenerative trajectories, with particular attention to post-SARS-CoV-2 neurological sequelae. By integrating these mechanistic insights, this review provides a conceptual foundation for the rational optimisation and bioengineering of MSC-EV-based therapies targeting convergent regulatory pathways in neurodegeneration.

## INTRODUCTION

Neurodegenerative diseases comprise a heterogeneous group of progressive disorders that, despite distinct genetic and environmental aetiologies, converge on shared pathological processes that ultimately compromise neuronal integrity and survival. Alzheimer’s disease (AD) is characterised by amyloid-β (Aβ) deposition, tau hyperphosphorylation and early synaptic collapse underlying cognitive decline^[1]^. Parkinson’s disease (PD) is defined by degeneration of nigrostriatal dopaminergic neurons together with α-synuclein pathology, leading to progressive motor and non-motor impairments^[2]^. Amyotrophic lateral sclerosis (ALS) involves the selective loss of upper and lower motor neurons, frequently associated with aggregation of TAR DNA-binding protein 43 (TDP-43), fused in sarcoma (FUS), or mutant superoxide dismutase (SOD1)^[3]^, while Huntington’s disease (HD) results from toxic gain-of-function effects of mutant huntingtin that drive striatal and cortical vulnerability^[4]^. Multiple sclerosis (MS), although classically viewed as an inflammatory demyelinating disorder, also features chronic axonal loss and compartmentalised central nervous system (CNS) inflammation that contribute to irreversible neurodegeneration^[5]^.

Despite these disease-specific features, AD, PD, ALS, MS and HD converge mechanistically on a conserved set of pathological processes, including chronic neuroinflammation with blood-brain barrier (BBB) dysfunction, oxidative and mitochondrial stress, impaired proteostasis with toxic protein aggregation, and progressive synaptic dysfunction accompanied by loss of neurotrophic support^[6]^. These processes form a tightly interconnected pathogenic network in which dysfunction in one domain amplifies injury in others, rendering neurodegeneration inherently multifactorial and self-reinforcing. This biological complexity complicates therapeutic intervention and helps explain why, despite decades of intensive research, available treatments remain largely symptomatic and exert limited impact on upstream degenerative mechanisms. Even highly targeted disease-modifying strategies, such as anti-amyloid monoclonal antibodies in AD, illustrate this mismatch by addressing a single pathological feature while leaving parallel degenerative cascades incompletely modified at the clinical level^[7]^. Collectively, these limitations underscore the need for multi-target, network-level therapeutic strategies capable of engaging the interconnected biology of neurodegeneration.

In this context, mesenchymal stromal/stem cells (MSCs) have emerged as promising candidates for neurodegenerative disease therapy due to their ability to modulate multiple pathogenic nodes simultaneously. Preclinical studies and early-phase clinical trials have demonstrated that MSCs exert potent immunomodulatory, antioxidant and neuroprotective effects across diverse models of neurodegeneration^[8,9]^. Increasing evidence now indicates that these benefits are mediated predominantly through paracrine mechanisms, with extracellular vesicles (EVs) released by MSCs acting as key effectors of intercellular communication^[10]^. This shift in understanding has redirected therapeutic interest from the cells themselves toward the MSC secretome, and MSC-derived EVs (MSC-EVs) in particular, as the principal drivers of MSC-mediated neuroprotection.

EVs are lipid bilayer-enclosed nanoparticles that transport bioactive cargo, including proteins, lipids, mRNAs, and non-coding RNAs, and influence recipient cells via intercellular transfer of this cargo. Although EVs are commonly subdivided into exosomes, microvesicles, and apoptotic bodies based on their biogenesis and size, current isolation and characterisation technologies do not reliably distinguish these subpopulations. In line with the recommendations of the Minimal Information for Studies of Extracellular Vesicles (MISEV) 2018 and 2023 guidelines, this review therefore adopts the inclusive term “EVs” to reflect current consensus and methodological limitations^[11,12]^.

Compared with their parent cells, MSC-EVs offer several practical and biological advantages for clinical translation in neurological disease. They exhibit low immunogenicity, avoid risks associated with cell engraftment, uncontrolled proliferation or vascular occlusion, and demonstrate favourable stability during storage and handling. Importantly, MSC-EVs have been shown in multiple experimental models to influence CNS pathology, either through direct CNS access or via modulation of neurovascular and immune interfaces, supporting their therapeutic relevance in CNS-targeted interventions^[8,9]^. In addition, MSC-EVs provide a scalable and more controllable therapeutic format than live cell products, enabling defined dosing, batch-to-batch standardisation, and greater compatibility with pharmaceutical quality control frameworks^[13]^. Together, these features position MSC-EVs as a compelling cell-free therapeutic platform in regenerative neurology, with growing application across experimental models of AD, PD, ALS, MS and HD^[8,9]^.

Despite a rapidly expanding body of literature reporting beneficial effects of MSC-EVs in neurodegenerative conditions, the molecular mechanisms underlying these effects remain poorly understood. Many studies rely on descriptive outcome measures, limiting mechanistic insight into how specific EV cargos engage disease-relevant cellular pathways. To address this gap, this review synthesises mechanistic evidence from experimental models to develop an integrated framework describing how MSC-EVs can modulate core, interconnected neurodegenerative modules. These include neuroinflammation with associated BBB dysfunction, oxidative and mitochondrial stress, impaired proteostasis, and disrupted neurotrophic signalling. Within each module, we examine how defined MSC-EV cargos influence recipient cell signalling and function across disease contexts, with the aim of informing more effective, multi-target therapeutic strategies for neurodegenerative disease.

Beyond classical neurodegeneration, emerging evidence suggests that viral infections can act as environmental triggers that engage the same pathogenic pathways, potentially accelerating or unmasking neurodegenerative processes in susceptible individuals^[14,15]^. We therefore extend this mechanistic framework to virus-associated neurodegeneration, using SARS-CoV-2 as a contemporary case to explore how sustained neuroinflammation, redox imbalance and disturbances in protein homeostasis following infection may contribute to long-term neurological vulnerability. Viewed through this lens, MSC-EV-mediated modulation of shared pathogenic pathways may offer opportunities not only for disease modification in established neurodegeneration, but also for early neuroprotective intervention in individuals with post-viral neurological sequelae (neuro-PASC or long COVID) at risk for neurodegeneration.

## MECHANISTIC MODULES OF MSC-EV ACTION IN NEURODEGENERATION

The multifactorial and interconnected nature of neurodegenerative disease raises a central mechanistic challenge: how a therapeutic intervention can meaningfully engage multiple pathogenic processes in parallel. MSC-EVs are uniquely positioned in this regard, as they deliver a complex repertoire of bioactive cargo, including non-coding RNAs, proteins, lipids and enzymes, that collectively modulate diverse cellular pathways rather than isolated molecular targets. Across experimental models of AD, PD, ALS, MS and HD, MSC-EV-mediated effects consistently converge on conserved pathological processes, reflecting a mode of action that is inherently pleiotropic yet biologically structured. To facilitate mechanistic interpretation of this complexity, this review adopts a modular analytical framework that organises overlapping MSC-EV effects into recurring pathological themes. This framework is designed to disentangle complex EV-mediated effects while recognising the tight biological coupling between processes *in vivo*. In the following subsections, we examine how MSC-EV cargo targets three interrelated modules: (1) neuroinflammation with associated BBB dysfunction; (2) oxidative and mitochondrial stress; (3) proteostasis failure with accompanying neurotrophic dysfunction. Together, these modules provide a unified mechanistic basis for understanding how MSC-EVs exert therapeutic effects across neurodegenerative disease contexts.

### Neuroinflammation and BBB dysfunction

Neuroinflammation and BBB dysfunction form an interdependent pathogenic module across AD, PD, ALS, and MS, with growing evidence supporting their involvement in HD. Chronic activation of microglia and astrocytes drives persistent release of inflammatory mediators such as tumour necrosis factor alpha (TNF‑α), interleukin (IL)‑1β, and IL‑6, leading to secondary damage of neurons, oligodendrocytes, and vascular cells^[16]^. In parallel, endothelial cells, pericytes, and astrocytic endfeet downregulate tight-junction proteins such as claudin-5, occludin, and zonula occludens (ZO)-1, while upregulating matrix metalloproteinases (MMP-2 and MMP-9), leading to increased vascular permeability and immune-cell infiltration^[16]^. These processes reinforce one another, establishing a self-amplifying loop in which persistent glial activation and BBB breakdown jointly drive immune dysregulation, parenchymal damage, and accelerated neurodegeneration.

Mounting evidence indicates that MSC-EVs may intervene within this cycle by reprogramming glial inflammatory states. In AD mouse models, treatment of MSC-EVs derived from umbilical cord (UC) and bone marrow (BM) reduced microgliosis and astrogliosis, decreased pro-inflammatory cytokine levels, and promoted a shift toward more homeostatic microglial phenotypes^[17,18]^. *In vitro*, adipose tissue (AT)-derived MSC-EVs were efficiently internalised by microglia and attenuated Aβ-induced activation by suppressing nitric oxide production and inflammatory mediators^[19]^. Comparable immunomodulatory effects are observed in ALS cellular models, where AT- and BM-MSC-EV treatment lowered nucleotide-binding oligomerisation domain (NOD)-like receptor family pyrin domain containing 3 (NLRP3) inflammasome activation and pro-inflammatory cytokines [TNF-α, IL-1β, IL-6, C-C motif chemokine ligand 2 (CCL2)], while restoring IL-10 expression in astrocytes derived from SOD1^G93A^ mice^[20,21]^. Consistent with this mechanism, induced pluripotent stem cells (iPSC)-derived MSC-EVs attenuated neuroinflammation by targeting NLRP3 via EV-associated miR-223-3p in AD mouse models^[22]^. Similarly, suppression of NLRP3 inflammasome activation has been shown to markedly protect dopaminergic neurons in the 1-methyl-4-phenyl-1,2,3,6-tetrahydropyridine (MPTP) mouse model of PD, highlighting the relevance of this pathway across neurodegenerative contexts^[23]^. Evidence from human *in vitro* systems further supports this regulatory role. In interferon (IFN)-γ/TNF-α-stimulated human microglial cell lines, BM-MSC-EV exposure normalised microglial morphology and shifted inflammatory and metabolic proteomic profiles toward a less activated, more homeostatic state^[24]^. Together, these findings support a model in which MSC-EVs may recalibrate the activation threshold of glial cells and constrain maladaptive inflammatory responses [Figure 1]^[25-27]^.

**Figure 1:**
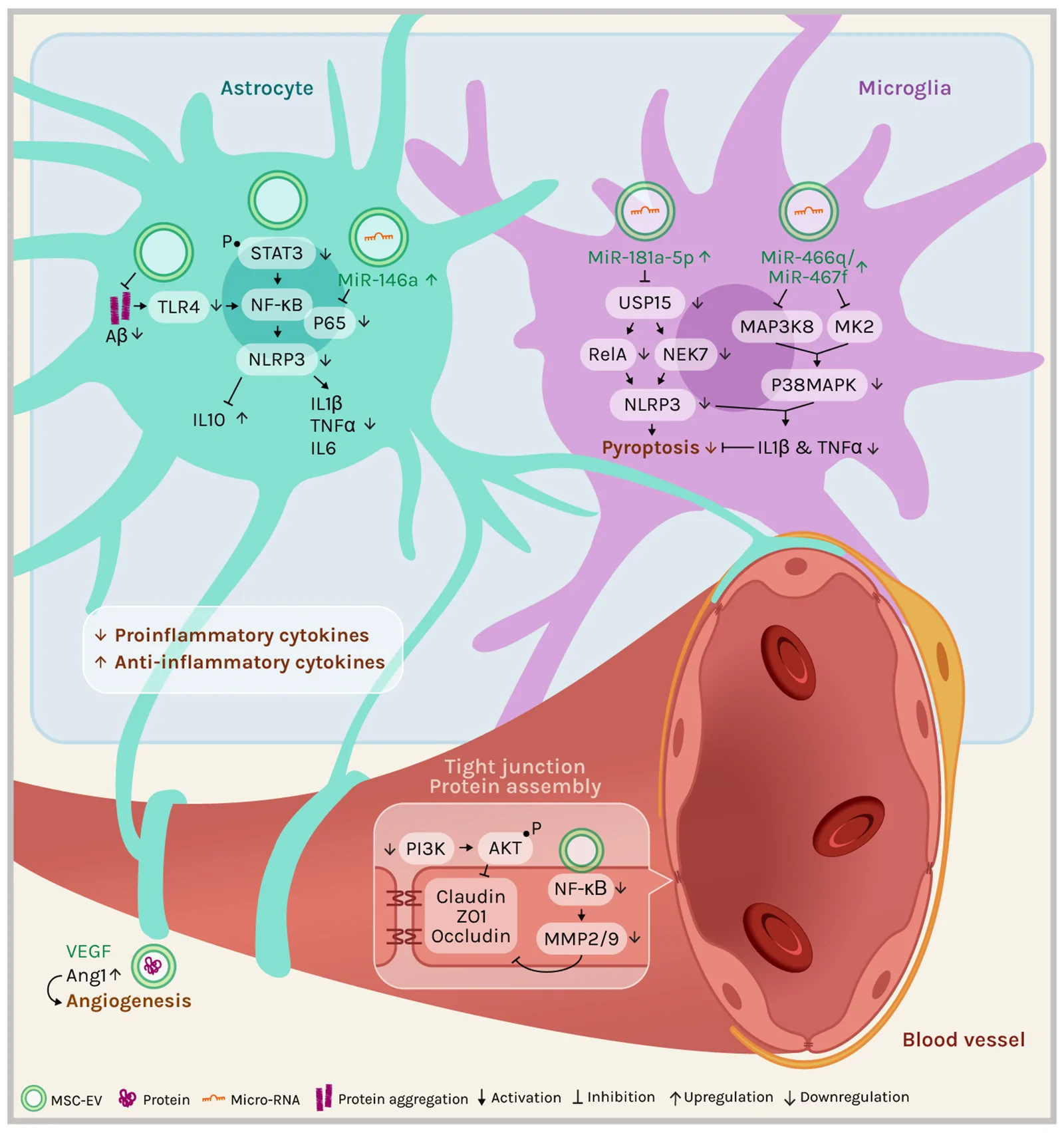
MSC-EV-mediated modulation of neuroinflammation and stabilisation of the BBB. Schematic illustrates a mechanistic framework in which MSC-EVs modulate a coupled neuroinflammatory-neurovascular circuit that is commonly disrupted across neurodegenerative diseases. Following uptake by activated astrocytes and microglia, MSC-EV cargo suppresses key inflammatory signalling programmes (including NF-κB and MAPK), resulting in reduced production of pro-inflammatory cytokines and a shift toward a more homeostatic glial state^[25]^. This attenuation of glial reactivity limits NLRP3 inflammasome activation and inflammatory cell stress responses, thereby dampening self-reinforcing inflammatory loops within the CNS. In parallel, MSC-EVs act on the neurovascular compartment to stabilise CNS barrier architecture by preserving tight-junction organisation and reducing MMP2/9 activity^[26,27]^. Through coordinated actions on glial inflammatory output and vascular integrity, MSC-EVs interrupt pathogenic feedback between neuroinflammation and barrier dysfunction, positioning EV cargo as an active regulator of neuroimmune-vascular homeostasis. MSC-EV: Mesenchymal stem cell-derived extracellular vesicle; BBB: blood-brain barrier; NF-κB: nuclear factor kappa-light-chain-enhancer of activated B cells; MAPK: mitogen-activated protein kinase; NLRP3: NOD-like receptor family pyrin domain containing 3; CNS: central nervous system; MMP-2/9: matrix metalloproteinase 2/9; TLR4: Toll-like receptor 4; ZO-1: zonula occludens-1; TJP1: tight junction protein 1; STAT3: signal transducer and activator of transcription 3; IL-10: interleukin-10; IL-1β: interleukin-1 beta; TNF-α: tumour necrosis factor alpha; IL-6: interleukin-6; Aβ: amyloid-beta; USP15: ubiquitin-specific protease 15; NEK7: NIMA-related kinase 7; p38 MAPK: p38 mitogen-activated protein kinase; PI3K: phosphatidylinositol 3-kinase; AKT: protein kinase B; VEGF: vascular endothelial growth factor; Ang-1: angiopoietin-1; MAP3K8: mitogen-activated protein kinase kinase kinase 8; MK2: mitogen-activated protein kinase-activated protein kinase 2.

This functional reprogramming of glial inflammatory responses is underpinned, at least in part, by specific EV-derived regulatory RNAs that target conserved inflammatory signalling pathways. In experimental autoimmune encephalomyelitis (EAE) mice, BM-MSC-EVs naturally containing miR-181a-5p suppressed microglial inflammation and pyroptosis by targeting ubiquitin-specific protease 15 (USP15) and modulating the p65 subunit (RelA)/NIMA-related kinase 7 (NEK7) axes, resulting in reduced IL-1β and IL-18 production, decreased demyelination, and improved clinical scores^[28]^. In ALS, IFN-γ-primed BM-MSC-EVs delivering miR-467f and miR-466q inhibited p38 mitogen-activated protein kinase (MAPK) signalling by targeting mitogen-activated protein kinase kinase kinase 8 (Map3k8) and Mk2, thereby reducing TNF-α and IL-1β secretion in both N9 microglia and primary microglia from SOD1^G93A^ mice^[29]^. In an AD *in vitro* model, BM-MSC-EV-associated miR-146a attenuated astrocytic neuroinflammation by inhibiting nuclear factor kappa-light-chain-enhancer of activated B cells (NF-κB) signalling in response to Aβ exposure^[30]^. Notably, hypoxia-conditioned MSC-EVs have been shown to dampen inflammatory signalling in AD mouse brains by reducing signal transducer and activator of transcription 3 (STAT3) phosphorylation and preventing nuclear translocation of NF-κB p65 in glial cells, demonstrating modulation of key inflammatory transcriptional regulators [Box 1]^[10,31-33]^. These findings indicate that MSC-EV miRNAs converge on shared inflammatory pathways, including NF-κB, MAPK, and inflammasome pathways across disease contexts, enabling coordinated suppression of maladaptive glial activation [Figure 1].

**Box 1:**
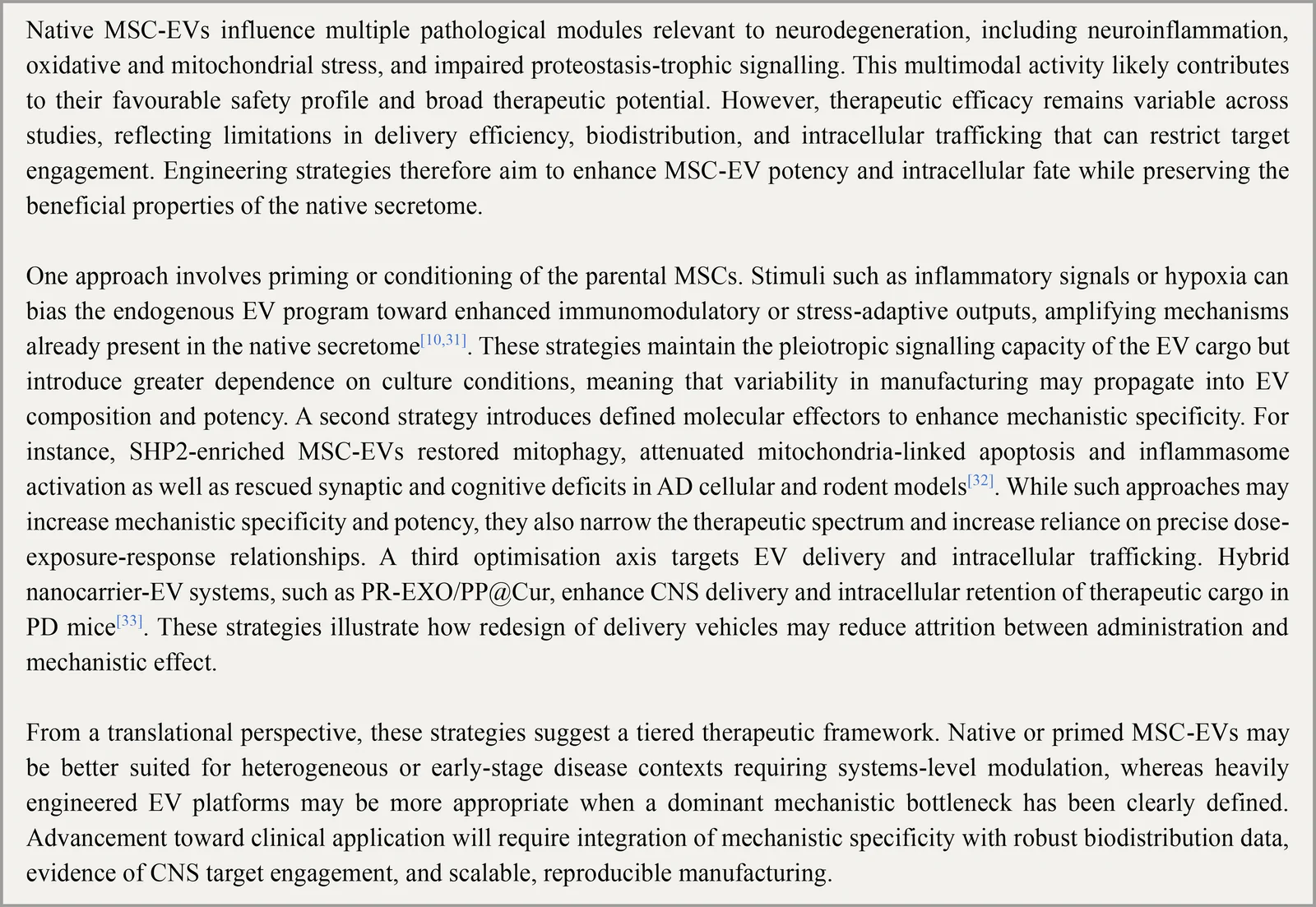
Engineering strategies to enhance MSC-EV therapeutic efficacy. AD: Alzheimer’s disease; CNS: central nervous system; EVs: extracellular vesicles; MSCs: mesenchymal stromal cells; PD: Parkinson’s disease; SHP2: Src homology region 2-containing protein tyrosine phosphatase 2.

Because sustained neuroinflammation directly compromises vascular integrity, effective immunomodulation is inseparable from stabilisation of the neurovascular unit. A growing body of evidence demonstrates that MSC-EVs exert direct protective effects on BBB structure and function. In EAE models, AT-MSC-EVs have been shown to inhibit C-X-C motif chemokine ligand 12 (CXCL12)-induced, integrin-dependent adhesion of T cells to endothelial cells via interactions with intercellular adhesion molecule (ICAM)-1 and vascular cell adhesion protein (VCAM)-1 *in vitro*^[34]^. Consistent with this mechanism, MSC-EV treatment markedly reduced rolling and firm arrest of encephalitogenic T cells within spinal cord microvessels *in vivo*, thereby limiting immune-cell entry into inflamed CNS tissue^[34]^. In context of CNS barrier dysfunction, UC-MSC-EV administration increased expression of tight-junction proteins such as claudin-5 and occludin, and reduced MMP-2 and MMP-9 expression^[27]^. *In vitro*, BM-MSC-EVs also modulate human brain pericytes, altering morphology and cytokine secretion profiles in a manner that depends on the inflammatory priming state of the parent MSCs, further supporting a role for MSC-EVs in tuning neurovascular inflammatory signalling^[35]^.

BBB protection further extends to endothelial resilience under proteotoxic and inflammatory stress. In an α-synuclein aggregate-induced BBB disruption model relevant to PD, UC-MSC-EVs preserved endothelial barrier integrity, reduced TNF-α induction, and interfered with aggregate-membrane interactions^[36]^. *In vivo*, neuroinflammatory regions in PD are characterised by elevated CCL2 expression, which promotes C-C motif chemokine receptor (CCR2)-dependent recruitment of peripheral immune cells to the substantia nigra. Leveraging this chemokine axis, MSC-EVs engineered to overexpress CCR2 preferentially accumulated in CCL2-rich inflamed regions in PD mouse models. This targeted localisation was accompanied by reduced peripheral immune-cell infiltration, together with attenuation of microglial activation and pro-inflammatory cytokine release^[37]^. The anti-inflammatory effects of MSC-EVs are reinforced by their protein cargo, which includes vascular endothelial growth factor (VEGF), fibroblast growth factor (bFGF), and angiopoietin (Ang)-1, molecules known to regulate vascular stability and immune signalling^[38,39]^. These proteins complement miRNA-mediated modulation of inflammatory and extracellular matrix pathways [Figure 1], suggesting that MSC-EV cargo may act through coordinated molecular programmes rather than isolated signalling events. Through this multi-layered cargo architecture, MSC-EVs can simultaneously influence endothelial survival, inflammatory signalling, and extracellular matrix dynamics.

Taken together, current evidence supports a mechanistic framework in which MSC-EVs suppress neuroinflammation and restore BBB functionality by targeting conserved signalling nodes that couple glial activation to vascular dysfunction. By interrupting reinforcing loops between inflammatory signalling, immune-cell trafficking, and barrier breakdown, MSC-EVs function as active molecular modulators rather than passive trophic agents. Nevertheless, much of the supporting evidence derives from rodent and *in vitro* systems, and critical gaps remain regarding cell-type-specific EV uptake, dose-response relationships, and temporal requirements for intervention. Addressing these gaps through standardised EV characterisation, longitudinal biomarker profiling of neuroinflammatory and BBB endpoints, and validation in models with higher translational fidelity will be essential to refine this mechanistic framework and guide the rational clinical development of MSC-EV-based therapies.

### Oxidative stress and mitochondrial dysfunction

Neurodegenerative diseases share a pathological module characterised by the tight interdependence of oxidative stress and mitochondrial dysfunction. Under physiological conditions, mitochondria maintain redox homeostasis through tightly regulated reactive oxygen species (ROS) production coupled to adenosine triphosphate (ATP) synthesis and antioxidant buffering. Disruption of this balance leads to excessive ROS accumulation, which damages mitochondrial DNA, lipids, and respiratory chain complexes, further impairing oxidative phosphorylation^[40]^. Compromised mitochondrial respiration, in turn, amplifies ROS generation, establishing a self‑reinforcing loop of redox imbalance and bioenergetic failure. This coupling is particularly detrimental in neurons, where high energetic demand, limited antioxidant reserves, and extended cellular architecture restrict compensatory responses. The manifestation of this shared redox-mitochondrial module is shaped by disease- and cell type-specific pathological drivers. In PD, dopaminergic neurons are particularly vulnerable as they rely heavily on mitochondrial oxidative phosphorylation and generate high basal levels of reactive oxygen species because of dopamine metabolism, making them sensitive to mitochondrial impairments^[41]^. In AD, mitochondrial dysfunction is tightly coupled to proteotoxic stress, as Aβ and tau directly disrupt mitochondrial dynamics, trafficking, and respiratory function, particularly at synapses^[42]^. In ALS, impaired ROS detoxification and mitochondrial damage are prominent in motor neurons expressing mutant SOD1 and TDP-43^[43,44]^, while HD involves transcriptional dysregulation of mitochondrial biogenesis and energy metabolism^[45,46]^. In MS, chronic demyelination disrupts oligodendrocyte-axon metabolic coupling, depriving axons of essential metabolic support and rendering them vulnerable to energy failure, mitochondrial dysfunction, and oxidative injury^[47]^.

Across these contexts, MSC-EVs have emerged as regulators of this module by intervening at multiple, mechanistically linked control points that collectively decouple oxidative stress from mitochondrial collapse. Rather than acting as simple antioxidants, MSC-EVs deliver defined miRNA, protein, enzyme, and lipid cargo that suppress ROS amplification, restore antioxidant buffering, stabilise mitochondrial integrity and turnover, and thereby prevent mitochondrial stress from progressing toward irreversible neuronal loss [Figure 2]^[21,32,48-50]^.

**Figure 2:**
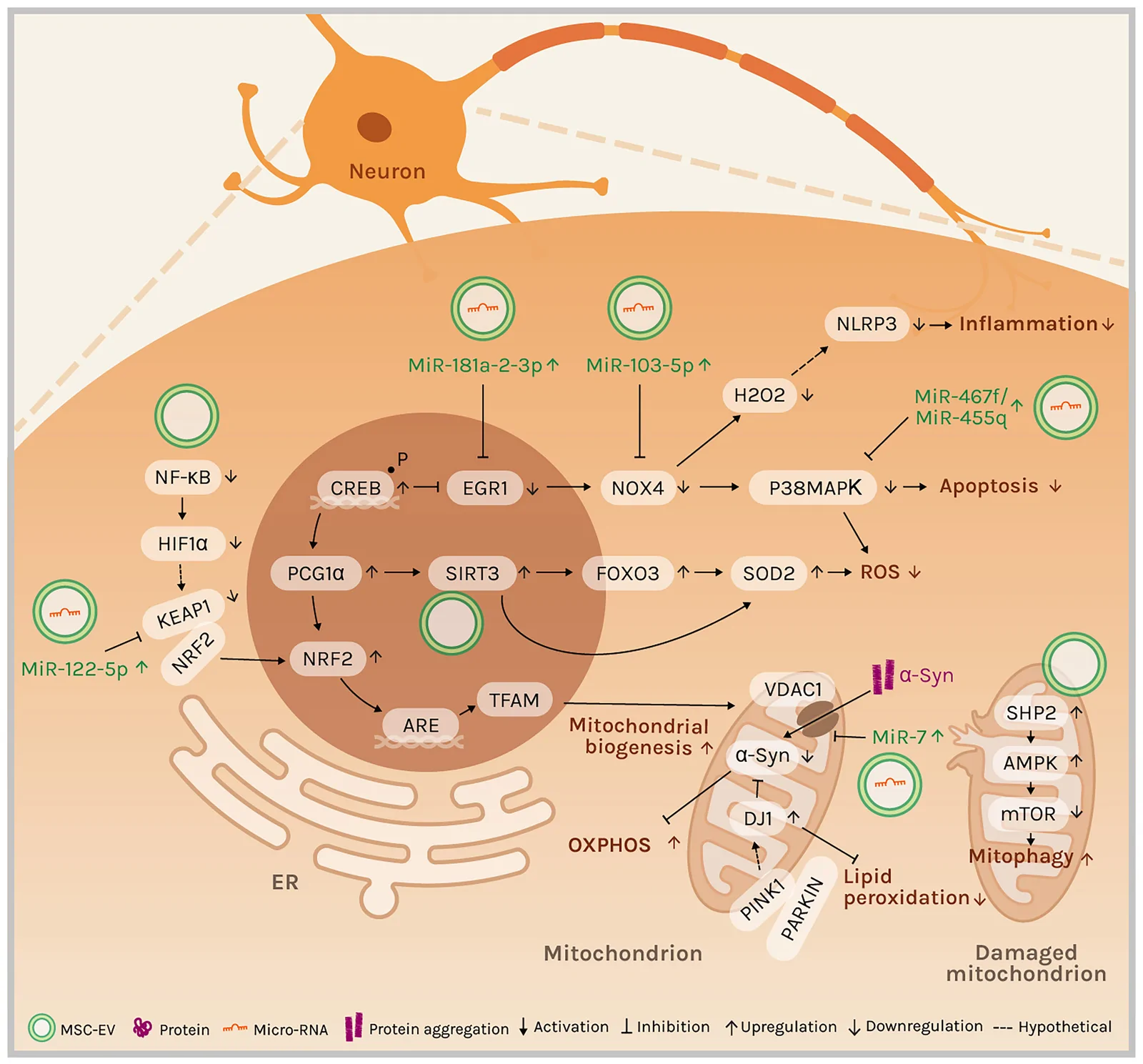
MSC-EV-mediated coordination of antioxidant defence and mitochondrial quality control. Schematic illustrates how MSC-EVs may regulate the coupled oxidative stress-mitochondrial dysfunction module that underlies neuronal vulnerability in neurodegenerative diseases. Following uptake by stressed neurons, MSC-EV cargo promotes nuclear localisation of NRF2, driving antioxidant gene transcription via ARE^[21]^. This response is coupled to upregulation of TFAM, supporting mitochondrial biogenesis^[48]^. In parallel, MSC-EV-associated miRNAs attenuate ROS amplification and apoptosis by modulating FOXO3- and MAPK-dependent stress signalling^[21]^. MSC-EV cargo further restricts mitochondrial accumulation of α-synuclein, preserving respiratory chain function and reducing lipid peroxidation^[49,50]^. At the level of organelle quality control, engineered MSC-EVs enhance neuronal mitophagy, facilitating the clearance of damaged mitochondria and reducing mitochondrial damage-associated apoptosis^[32]^. Together, these coordinated actions position MSC-EVs as integrative regulators of neuronal stress resilience, acting upstream to decouple oxidative stress from mitochondrial failure and prevent progression toward irreversible bioenergetic collapse. MSC-EV: Mesenchymal stem cell-derived extracellular vesicle; NRF2: nuclear factor erythroid 2-related factor 2; ARE: antioxidant response element; TFAM: mitochondrial transcription factor A; ROS: reactive oxygen species; FOXO3: forkhead box O3; MAPK: mitogen-activated protein kinase; α-syn: alpha-synuclein; NF-κB: nuclear factor kappa-light-chain-enhancer of activated B cells; HIF-1α: hypoxia-inducible factor 1 alpha; KEAP1: Kelch-like ECH-associated protein 1; CREB: cAMP response element-binding protein; PGC-1α: peroxisome proliferator-activated receptor gamma coactivator 1-alpha; EGR1: early growth response 1; SIRT3: sirtuin 3; SOD2: superoxide dismutase 2; NLRP3: NOD-like receptor family pyrin domain containing 3; H_2_O_2_: hydrogen peroxide; NOX4: NADPH oxidase 4; p38 MAPK: p38 mitogen-activated protein kinase; VDAC1: voltage-dependent anion channel 1; PINK1: PTEN-induced kinase 1; OXPHOS: oxidative phosphorylation; AMPK: AMP-activated protein kinase; mTOR: mammalian target of rapamycin; ER: endoplasmic reticulum.

A first regulatory axis involves suppression of ROS amplification and reinforcement of antioxidant defence through redox-sensitive transcriptional and signalling programs. In PD cellular and mouse models, UC-MSC-EVs delivering miR-181a-2-3p attenuated ROS accumulation and prevented dopaminergic neuron apoptosis by modulating early growth response-1 (EGR1) via inhibition of the nicotinamide adenine dinucleotide phosphate (NADPH) oxidase 4 (NOX4)-p38 MAPK pathway^[51]^. Similarly, trophoblast-derived MSC-EVs enriched in miR-100-5p directly downregulated NOX4, limiting ROS production and engaging the NOX4-nuclear factor erythroid 2-related factor 2 (NRF2) cascade to preserve nigrostriatal integrity and motor performance in MPTP mice^[52]^. In a 6-hydroxydopamine (6-OHDA) PD model, human umbilical cord (hUC)-MSC-EVs containing miR-7-5p, miR-125-5p, miR-126-3p, miR-122-5p, and miR-199-3p activated NRF2 signalling, reduced oxidative stress, restored dopamine levels, and improved behavioural outcomes^[53]^. Among these, miR-7 directly regulates α-synuclein expression, linking suppression of proteotoxic burden to reduced oxidative load^[54]^. Growth-factor cargo can further amplify this response, as brain-derived neurotrophic factor (BDNF)-loaded hUC-MSC-EVs suppressed 6-OHDA-induced apoptosis, activated NRF2, and improved dopaminergic neuron survival and cytoskeletal stability *in vitro*^[55]^. Similar redox-protective reprogramming has been reported in ALS, where BM-MSC-EV-associated miR-467f/466q attenuated p38-MAPK signalling and inflammatory mediator expression in SOD1^G93A^ microglia and astrocytes^[21,29]^. Collectively, these studies position MSC-EV cargo upstream of ROS accumulation, acting on amplification nodes [Figure 2].

Because damaged mitochondria are themselves a major source of secondary ROS, effective antioxidant control is inseparable from preservation of mitochondrial integrity and turnover. This reflects the rate-limiting role of mitochondrial stress in dopaminergic neuron survival. In APP/PS1 AD mice, MSC-EV treatment restored mitochondrial ultrastructure and normalised fission-fusion markers, including COX IV, Tom20, and FIS1, alongside rebalancing of NRF2 pathway components, underscoring reciprocal regulation between redox balance and organelle maintenance^[56]^. In PD rat models, UC-MSC-EVs promoted autophagy-dependent neuroprotection, rescuing dopaminergic neurons and normalising dopamine metabolism [3,4-Dihydroxyphenylacetic acid (DOPAC), homovanillic acid (HVA)] and motor behaviour^[57]^. In AD, engineered MSC-EVs containing tyrosine phosphatase-2 (SHP2) enhanced mitophagy, supporting selective clearance of damaged mitochondria as an adaptive survival response^[32]^ [Box 1]. Within this axis, mitochondrial deacetylase sirtuin 3 (SIRT3) emerges as a critical integrator of antioxidant defence and mitochondrial quality control. MSC-EV-mediated SIRT3 activation could potentially enhance SOD2-dependent mitochondrial antioxidant defence, thereby promoting mitochondrial resilience and protecting dopaminergic neurons from oxidative injury ^[58,59]^ [Figure 2].

In addition to transcriptional and metabolic regulation, MSC-EVs provide enzymatic antioxidant activity that operates on shorter timescales, buffering oxidative stress while longer-term adaptive programs are engaged. Wharton’s Jelly MSC-EVs were able to transfer active catalase to primary rat hippocampal neurons exposed to Aβ oligomers, reducing ROS and preventing synaptic loss^[60]^. MSC-EV treatment has been shown to enhance antioxidant capacity in recipient brain tissue, including increased MnSOD (SOD2) and glutathione peroxidases, enzymes capable of detoxifying superoxide and peroxides and thereby stabilising mitochondrial membranes and respiratory complexes^[61]^. These enzymatic defences act in parallel with transcriptionally regulated antioxidant systems, notably the NRF2-Keap1 pathway, which coordinates sustained glutathione synthesis and redox homeostasis [Figure 2]. In a rotenone-induced PD rat model, BM-MSC-EVs treatment increased Parkin and DJ-1 levels while reducing α-synuclein accumulation, changes consistent with improved mitochondrial and redox homeostasis^[50]^. Reports of MSC-EV-associated mitochondrial components, including mitochondrial transcription factor A (TFAM) and mitochondrial DNA (mtDNA) fragments, further raise the possibility that MSC-EVs contribute to bioenergetic support beyond redox regulation^[62]^. However, the extent to which such components functionally integrate into host mitochondrial networks, and the dose and context dependence of these effects, remain incompletely resolved and require systematic validation.

Collectively, current evidence supports a mechanistic framework in which MSC-EVs may mitigate oxidative stress and mitochondrial dysfunction through coordinated regulation of ROS amplification, antioxidant buffering, mitochondrial integrity and turnover, and enzymatic redox control. The predominance of PD models in this literature reflects both the central role of mitochondrial dysfunction in dopaminergic neuron survival and the experimental accessibility of well-established toxin-based models, rather than a selective responsiveness of PD to EV-based interventions. Importantly, EV-mediated modulation of mitochondrial stress response has also been reported across AD, ALS, HD, and MS. This recurring engagement of redox control, mitochondrial quality maintenance, and bioenergetic support suggests that MSC-EVs act on shared cellular stress pathways [Figure 2]. Refining this framework will require causal mapping of EV cargo-target interactions and functional mitochondrial outcomes across disease-relevant cell types, an essential step toward defining therapeutic windows and guiding the clinical translation of MSC-EV-based strategies.

### Impairment of proteostasis and neurotrophic signalling

Loss of proteostasis and neurotrophic signalling represent converging pathological mechanisms across neurodegenerative diseases, jointly undermining neuronal survival and repair. Disrupted protein quality control leads to the accumulation of toxic aggregates - including Aβ and tau in AD, α-synuclein in PD, mutant huntingtin (mHTT) in HD, and misfolded SOD1 and TDP-43 in ALS. These aggregates overwhelm proteasomal and autophagic systems, propagate cellular stress, and perturb trafficking and synaptic function. Concurrently, declining neurotrophic input, especially BDNF, glial cell line-derived neurotrophic factor (GDNF), and insulin-like growth factor-1 (IGF-1), reduces synaptic maintenance and regenerative capacity, lowering the threshold for proteotoxic injury^[63,64]^. These processes are mutually reinforcing, as proteostatic collapse impairs growth-factor responsiveness while trophic withdrawal weakens autophagy and stress-response programs. Therapeutic strategies that restore protein homeostasis while re-engaging trophic signalling therefore have strong potential for disease modification.

MSC-EVs appear well positioned to target this coupled pathological module. Current evidence suggests that MSC-EVs influence proteostasis through three interrelated mechanisms: enhancing degradative clearance of aggregation-prone proteins, limiting upstream drivers of pathogenic protein production, and stabilising the intracellular protein-folding environment [Figure 3]^[65,66]^. These actions simultaneously support neurotrophic signalling and synaptic integrity.

**Figure 3:**
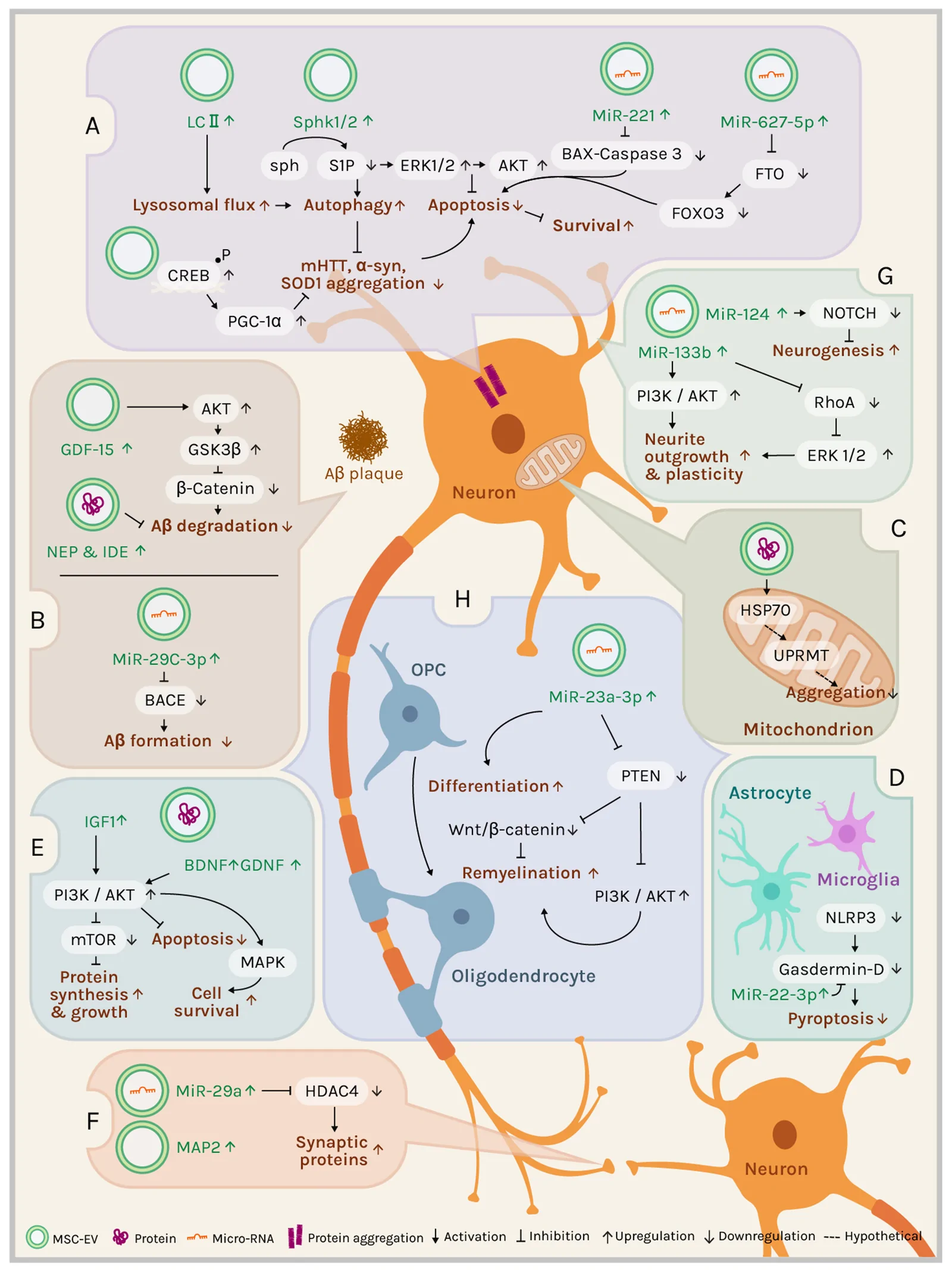
MSC-EVs reprogram proteotoxic stress toward neurotrophic recovery. Schematic illustrates how MSC-EVs act across multiple, interconnected layers of the proteotoxic cascade while simultaneously reinforcing neurotrophic and regenerative signalling. At the level of aggregate burden, MSC-EV cargo promotes clearance of pathogenic proteins, including Aβ, α-synuclein, mutant SOD1 and mHTT, through activation of intracellular degradation pathways. (A) This includes engagement of autophagic and lysosomal flux as well as enhancement of proteolytic capacity; (B) thereby reducing aggregate accumulation and associated cellular death. In parallel, MSC-EV-associated regulatory cargo modulates signalling pathways that reduce amyloidogenic processing and reinforce chaperone (HSP70)-mediated folding responses and restrain stress-induced apoptotic and pyroptotic cell death^[65]^ (C and D). These proteostatic effects are closely coupled to activation of survival and metabolic transcriptional programmes that support neuronal viability under chronic stress. Beyond aggregate handling, MSC-EVs deliver neurotrophic signals that re-engage growth, plasticity, and repair pathways in neurons and glial cells. Growth factors and regulatory miRNAs converge on PI3K/Akt and MAPK signalling to promote neurite outgrowth (E); synaptic protein expression (F); cytoskeletal stability (G); and network function. In OPCs, MSC-EV cargo further supports differentiation and remyelination, indirectly reinforcing long-term proteostatic balance^[66]^ (H). MSC-EV: Mesenchymal stem cell-derived extracellular vesicle; Aβ: amyloid-beta; SOD1: superoxide dismutase 1; mHTT: mutant huntingtin; HSP70: heat shock protein 70; UPRMT: mitochondrial unfolded protein response; PI3K: phosphatidylinositol 3-kinase; AKT: protein kinase B; MAPK: mitogen-activated protein kinase; OPC: oligodendrocyte progenitor cell; LC3-II: microtubule-associated protein 1 light chain 3-II; SphK: sphingosine kinase; S1P: sphingosine-1-phosphate; ERK: extracellular signal-regulated kinase; GDF-15: growth differentiation factor 15; GSK-3β: glycogen synthase kinase 3 beta; NEP: neprilysin; IDE: insulin-degrading enzyme; BACE1: beta-site amyloid precursor protein cleaving enzyme 1; IGF-1: insulin-like growth factor 1; BDNF: brain-derived neurotrophic factor; GDNF: glial cell line-derived neurotrophic factor; mTOR: mammalian target of rapamycin; HDAC4: histone deacetylase 4; MAP2: microtubule-associated protein 2; NLRP3: NOD-like receptor family pyrin domain containing 3; NOTCH: Notch receptor; RhoA: Ras homolog family member A; ERK1/2: extracellular signal-regulated kinase 1/2; PTEN: phosphatase and tensin homolog; AMPK: AMP-activated protein kinase.

A substantial body of evidence indicates that MSC-EVs facilitate clearance of toxic protein aggregates by delivering proteolytic enzymes and activating intracellular degradation pathways. In AD rodent models, EVs derived from AT- and UC- MSCs delivered active neprilysin (NEP) and insulin-degrading enzyme (IDE), reducing Aβ and improving cognition in APP/PS1 mice^[17,67]^. MSC-EVs can also reinforce aggregate clearance by activating signalling programmes that upregulate endogenous proteolytic capacity. BM-MSC-EVs enriched in growth differentiation factor-15 (GDF-15) increased NEP and IDE expression in Aβ42-induced SH-SY5Y cells through activation of the protein kinase B (AKT)/glycogen synthase kinase 3 beta (GSK-3β)/β-catenin pathway, a signalling axis known to regulate transcriptional programmes involved in protein turnover and neuronal survival^[68]^. In parallel, sphingosine kinase (SphK)/sphingosine-1-phosphate (S1P) signalling has been shown to enhance degradation of Aβ42 aggregates and preserve synaptic structure and function in AD mice^[69]^. Notably, most mechanistic studies of MSC-EV-mediated proteostasis remain focused on Aβ pathology. This represents an important limitation, as tau hyperphosphorylation, aggregation, and intercellular propagation correlate more closely with clinical progression in AD than amyloid burden. Whether MSC-EVs can meaningfully influence tau pathology therefore remains insufficiently resolved.

In parallel with protease-mediated clearance, MSC-EVs enhance intracellular autophagy pathways that enable removal of aggregation-prone proteins. In PD cellular and rodent models, UC-MSC-EV treatment induced autophagic flux by increasing microtubule-associated protein 1 light chain 3-II (LC3-II) levels, facilitated α-synuclein clearance, reduced dopaminergic neuron loss and improved motor outcomes^[57]^. 6-OHDA-stimulated SH-SY5Y neuroblastoma cells show that UC-MSC-EVs activate autophagy via AMP-activated protein kinase (AMPK)-mammalian target of rapamycin (mTOR) signalling, thereby reducing apoptotic stress. These effects are recapitulated *in vivo*, where MSC-EV administration preserves dopaminergic neurons, restores dopamine and its metabolites (DOPAC and HVA), and improves motor performance in PD rat models^[33]^. Engineering approaches can amplify these effects, as a hybrid nanocarrier combining MSC-EVs with curcumin (PR-EXO/PP@Cur) reduced α-synuclein oligomers and preserved motor function more effectively than naïve EVs^[33]^ [Box 1]. At present, it remains unclear whether this benefit reflects enhanced delivery of curcumin alone or synergy with MSC-EV-mediated proteostatic signalling.

Evidence from ALS and HD models suggests that MSC-EVs may also stabilise broader neuronal stress-response programs. In SOD1^G93A^ neuronal models of ALS, AT-MSC-EVs reduced mutant SOD1 aggregation and restored cAMP response element-binding protein (CREB) phosphorylation and peroxisome proliferator-activated receptor gamma coactivator 1-alpha (PGC-1α) signalling, linking improved proteostasis to mitochondrial support and pro-survival transcriptional programmes^[70]^. Consistent with this, our proteomic study in iPSC-derived motor neurons carrying C9ORF72, TDP-43, or FUS mutations identified both shared and mutation-specific ALS signatures and showed that BM-MSC-EVs could reverse proteomic abnormalities across genetic backgrounds, with a functional effect detected in FUS motor neurons *in vitro*^[71]^. Beyond aggregate handling, MSC-EVs also exert regenerative effects on neuronal structure. In SOD1^G93A^ primary motor neurons, MSC-EV treatment promoted axonal growth, accompanied by enrichment of miRNAs (miR-221-3p, miR-22-3p, miR-125b-3p, miR-145-5p) linked to neuronal differentiation and cytoskeletal regulation^[72,73]^. Additionally, an independent study demonstrated that MSC-EV-delivered miR-22 can suppress neuronal pyroptosis by targeting gasdermin-D, thereby preventing inflammatory cell death and indirectly preserving intracellular proteostasis under chronic stress conditions in AD mice^[74]^. Together, these findings suggest that MSC-EVs may shift neurons toward a more resilient, repair-competent state [Figure 3].

Beyond promoting degradative clearance, MSC-EVs may also act upstream by suppressing pathogenic protein production, thereby complementing downstream proteolytic and autophagic mechanisms. EV-delivered miR-29c-3p represses beta-site amyloid precursor protein cleaving enzyme 1 (BACE1) via Wnt/β-catenin signalling, thereby reducing amyloidogenic processing^[75]^. Additional gene-regulatory strategies have also been reported. For example, engineered MSC-EVs delivering a small interfering RNA targeting fat mass and obesity-associated protein (FTO), an N^6^-methyladenosine (m6A) RNA demethylase implicated in neuronal stress responses, were reported to reduce dopaminergic cell death, potentially via miR-627-5p^[76]^. Although promising, such approaches require careful validation of cargo specificity and downstream regulatory networks.

A further mode of MSC-EV action involves stabilising the protein-folding environment through delivery of molecular chaperones and reinforcement of cellular stress-response pathways. Wharton jelly-derived MSC-EVs contain heat shock proteins such as Hsp70^[77]^. Hsp70 has been shown to reduce HTT aggregation in cellular HD models, raising the possibility that EV-associated chaperones may influence aggregate burden ^[78,79]^. These proteostatic mechanisms likely interact with inflammatory and redox pathways, as suppression of oxidative injury and inflammatory cell death can preserve proteostasis under chronic stress.

MSC-EVs may further contribute to neuronal resilience by reinforcing neurotrophic signalling pathways that support survival, synaptic maintenance, and plasticity. This effect is mediated both through delivery of miRNAs and diverse growth factors, including BDNF, GDNF, IGF-1, and VEGF^[39]^. For example, Wharton’s jelly-derived MSC-EVs enriched in miR-29a downregulated histone deacetylase 4 (HDAC4), an epigenetic regulator aberrantly elevated in AD mouse brains, restoring expression of synaptic genes such as Homer1 and Syn2 and improving synaptic plasticity^[77]^. Consistent with this, UC-MSC-EV treatment in 6-OHDA models increased microtubule-associated protein 2 (MAP2) expression and reduced hyperphosphorylated tau, indicating improved cytoskeletal stability and neuronal integrity^[57]^. Beyond these effects, MSC-EV cargo can directly engage pathways controlling neuronal differentiation and structural plasticity. MSC-EV-mediated delivery of miR-133b, which is markedly downregulated in PD, has been shown to promote neurite outgrowth and neural plasticity through suppression of RhoA and activation of extracellular signal-regulated kinase 1/2 (ERK1/2) and phosphatidylinositol 3-kinase (PI3K)/Akt signalling, while also regulating Pitx3, a transcription factor critical for dopaminergic neuron development and maintenance^[80,81]^. Similarly, MSC-EVs carrying miR-124 and miR-145 have been shown to drive maturation of human neural progenitors and increase glutamate transporter expression in neurons and astrocytes, supporting functional network integration and excitatory homeostasis^[82]^.

At the circuit level, MSC-EVs can translate these molecular effects into restoration of synaptic function. In APP/PS1 mice, MSC-EV treatment restored CA1 pyramidal neuron excitability disrupted by Aβ-associated calcium transient abnormalities. In AD mice, EVs from hypoxia-preconditioned MSCs reduced Aβ oligomers while increasing growth-associated protein-43 and synapsin-1 and improving memory performance, consistent with coupled effects on aggregate stress and synaptic repair^[31]^ [Box 1]. In oligodendrocyte progenitor cells (OPCs) derived from EAE mice, UC-MSC-EV delivery of miR-23a-3p activated T-box brain protein 1 (Tbr1)/Wnt and PI3K/Akt signalling to promote oligodendrocyte differentiation and myelin protein expression, indirectly preserving neuronal proteostasis by reducing axonal metabolic stress^[66]^.

Collectively, these findings support a unified mechanistic framework in which MSC-EVs restore proteostasis and neurotrophic support through tightly coupled actions: promoting autophagic and proteolytic clearance, suppressing pathogenic protein production, stabilising folding environments, and re-engaging survival and plasticity signalling. Rather than acting as passive carriers of growth factors, MSC-EVs function as dynamic regulators of neuronal resilience, integrating trophic signalling with protein quality control across disease contexts.

## DETERMINANTS OF MSC-EV THERAPEUTIC EFFICACY

The mechanistic modules outlined above define how MSC-EVs can engage key pathological processes in neurodegeneration. However, reported therapeutic outcomes vary substantially across studies. This variability is more likely to reflect differences in MSC-EV preparation and effective *in vivo* delivery to target tissues rather than inconsistency of the underlying biological mechanisms. Contributing factors include the tissue source of the parental MSCs, whether cells are primary or immortalised, culture and conditioning parameters, EV isolation and purification strategies (which influence co-purified proteins, lipoproteins, or soluble mediators), and dosing and delivery routes that determine biodistribution and persistence *in vivo*^[83,84]^. While many of these variables are not systematically controlled across studies, they collectively constrain therapeutic efficacy and complicate cross-study comparison. In this section, we focus on two determinants with the strongest mechanistic and empirical support: MSC tissue source as a driver of cargo bias, and biodistribution as a gatekeeper of target engagement, while recognising that EV purity and manufacturing consistency remain critical but under-addressed variables.

### MSC-EV cargo heterogeneity and source-dependent bias

MSC-EVs are increasingly investigated as therapeutics for neurodegenerative disease, yet how molecular cargo differences across MSC sources translate into distinct or overlapping therapeutic mechanisms remains poorly understood. Comparative proteomic and transcriptomic analyses reveal that MSC-EVs derived from BM, AT, and UC share a conserved set of paracrine factors enriched in immunomodulatory, antioxidant, and trophic factors, alongside differences in the relative abundance of specific cargo classes^[85-87]^. These differences reflect biological variation between tissue sources rather than discrete functional identities and are best interpreted as quantitative shifts in cargo composition that may influence the relative engagement of mechanistic axes.

Consistent with this view, BM-MSC-EVs show relative enrichment in proteins involved in intracellular trafficking, proteasome-associated machinery, as well as synaptic regulators such as ADAM10, ADAM9 and Notch2^[86]^. AT-MSC-EVs tend to show stronger representation of secretory and immunomodulatory pathways, and oxidative stress response programmes^[86,88]^. UC-MSC-EVs display higher relative abundance of proteins such as plasminogen activator inhibitor (PAI)-1 and 14-3-3ζ (YWHAZ), associated with Wnt signalling, extracellular matrix (ECM) remodelling and vascular integrity^[88,89]^. Importantly, these molecules are not exclusive to any single MSC source but differ in relative abundance, and their functional implications should therefore be considered probabilistic rather than deterministic.

Such cargo differences are often interpreted as aligning with distinct mechanistic emphases, as illustrated by studies in AD. In AD cellular and rodent models, MSC-EVs have most often been linked to synaptic maintenance and neuronal survival, consistent with their ability to restore synaptic plasticity‑related gene expression and improve cognitive deficits^[77]^. AT-MSC-EVs have been prominently associated with extracellular proteostasis through delivery of Aβ-degrading enzymes such as neprilysin, reducing amyloid burden *in vitro* and *in vivo* settings^[67]^. UC-MSC-EVs have frequently been studied in relation to immune and neurovascular interfaces, reprogramming microglia toward a more phagocytic state and promoting Aβ clearance *in vivo*, consistent with their receptor-, Wnt-, and ECM-related cargo^[86,89]^. However, these patterns largely reflect differences in experimental focus and study design rather than controlled source comparisons within identical models. As a result, they should be interpreted as mechanistic tendencies, not evidence of source superiority.

Despite quantitative differences in cargo composition, preclinical studies report broadly beneficial effects from MSC-EVs across sources^[90]^, consistent with the possibility that the shared paracrine core may be sufficient to confer therapeutic impact in commonly used preclinical animal models^[86,91]^. However, the field still lacks rigorous comparative studies controlling for MSC origin, EV manufacturing, dosing, route, and outcome measures, precluding designation of an optimal MSC source for neurodegenerative applications.

Taken together, available evidence indicates that MSC tissue source influences the relative abundance of bioactive proteins and regulatory RNAs packaged into EVs, thereby modulating the balance of pathways engaged in recipient cells. These differences do not constitute discrete therapeutic identities, nor do they justify designation of a superior source for neurodegenerative applications. Rather, they suggest that MSC origin modulates the balance of shared regulatory mechanisms within a conserved paracrine framework. The central implication is therefore not source selection as a binary choice, but recognition that tissue origin may fine-tune module engagement. Resolving whether such tuning is clinically meaningful will require systematic head-to-head comparisons conducted under harmonised manufacturing and delivery conditions.

### MSC-EV biodistribution and pharmacokinetics: implications for therapeutic engagement in neurodegeneration

The therapeutic efficacy of MSC-EVs in neurodegeneration depends not only on their cargo composition but also on whether EVs and/or their bioactive cargo achieve sufficient exposure at disease-relevant sites. Neurodegenerative disorders are spatially heterogeneous and characterised by dynamic neurovascular dysfunction, glial activation, and altered immune trafficking. In this context, administration route and pharmacokinetic behaviour primarily shape systemic clearance, peripheral sequestration, and the likelihood of CNS exposure. Although MSC-EV-associated signals have been detected beyond the BBB, their degree of CNS exposure is strongly influenced by vascular state, delivery route, and disease context^[90]^. Brain delivery constraints and methodological caveats in EV tracking have been reviewed extensively and remain central to interpreting the literature^[91]^.

Across studies, intravenous (IV) administration of EVs results in rapid clearance by the mononuclear phagocyte system, with dominant uptake in liver and spleen and only a minute fraction accessing the CNS^[92-94]^. Despite this limited brain accumulation, IV-delivered MSC-EVs consistently produce molecular and functional benefits in rodent models of AD, PD, ALS, and MS^[20,56,57,95]^. This mismatch between limited brain accumulation and measurable benefit challenges the assumption that widespread parenchymal penetration is a prerequisite for therapeutic efficacy. One explanation is that neurodegenerative pathology is associated with regional BBB dysfunction and neurovascular inflammation, which may increase permissiveness to circulating MSC-EVs compared with healthy brain. A second possibility is that many MSC-EV effects may occur at neurovascular and immune interfaces, where modulation of endothelial cells and perivascular macrophages can propagate secondary effects into neural networks without requiring extensive neuronal uptake^[96,97]^. Whether similar exposure-effect relationships hold in humans remains uncertain and defining dose-exposure-response relationships represents a key translational gap. Systemic MSC-EV administration, delivered as either single or repeated doses, has improved behavioural and disease-associated inflammatory and pathological outcomes in AD, PD, and ALS mice^[20,53,56]^, but whether this reflects greater CNS exposure, prolonged peripheral immunomodulation, or both is not yet resolved.

Intranasal (IN) administration has been explored as a non-invasive strategy to bypass hepatic clearance by exploiting olfactory and trigeminal pathways. In rodents, IN-delivered UC-MSC-EVs labelled via amine-reactive conjugation of EV surface proteins were detected in the hippocampus, cortex, and olfactory bulbs, and are taken up by neurons, astrocytes, and microglia^[98]^. Perets *et al.* used computed tomography combined with gold nanoparticle labelling and reported selective accumulation of IN-administered BM-MSC-EVs within inflamed or degenerating regions in AD and PD models, with detectable signal persisting for up to 96 h^[99]^. Moreover, functional studies reporting reductions in Aβ burden and neuroinflammation after IN administration often do not provide robust quantification of intact vesicle accumulation in brain parenchyma, despite observing behavioural improvement^[18,100]^. In EAE models, for example, signal has been reported predominantly in peripheral lymphoid tissues rather than CNS tissue^[101]^. Together, these data support functional benefit in rodent systems while leaving unresolved the extent to which therapeutic effects depend on direct parenchymal EV uptake versus indirect immune or neurovascular mechanisms.

However, translational scaling introduces substantial anatomical and physiological constraints in large animals. In pig-tailed macaques, IV-administered MSC-EVs labelled with a membrane-anchored bioluminescence reporter (palmGRET) exhibited longer circulation times and low but measurable cerebrospinal fluid (CSF) levels yet remained predominantly sequestered in liver and spleen^[102]^. The same study further demonstrated negligible nose-to-brain delivery following IN administration, with a substantial proportion of administered EVs recoverable from the nasal cavity^[102]^. These findings underscore mucociliary clearance and epithelial barriers as dominant constraints on CNS access in primates. Positron emission tomography (PET)/magnetic resonance imaging (MRI) studies in rhesus macaques using radio-labelled EVs showed that intrathecal (IT) administration achieved the highest CNS exposure, IV delivery produced low but detectable brain signal, and IN delivery resulted in negligible CNS distribution^[103]^. These findings indicate that rodent “nose-to-brain” efficiency does not scale directly to primates, likely due to differences in nasal anatomy, mucociliary clearance, and epithelial barriers. Whether therapeutically sufficient CNS exposure can be achieved through IN delivery in humans remains unresolved.

Interpretation of EV biodistribution data is further complicated by methodological limitations. Many rodent studies rely on lipophilic dyes such as PKH to visualise EV brain uptake. While useful for *in vitro* and short-term tracking, these dyes can transfer to host membranes or persist following EV degradation, confounding attribution of signal to intact EVs^[39,104]^. Radiolabelling and genetic reporters improve specificity but do not fully resolve whether detected signal represents intact vesicles, phagocytosed material, or redistributed cargo^[92]^. Moreover, most studies do not distinguish between the fate of the EV membrane and that of its functional cargo after cellular uptake. This distinction is particularly relevant in the CNS, where limited EV entry may still influence network-level inflammatory and metabolic signalling. Dose-exposure-response relationships remain poorly defined, with many studies employing supraphysiological EV doses without establishing minimal effective thresholds or persistence within CNS compartments. Immune responses add an additional layer of complexity, as repeated IV administration of xenogeneic EVs in macaques accelerates clearance^[102]^, an effect rarely quantified in rodent neurodegenerative models.

Taken together, current evidence supports three pragmatic conclusions. First, native MSC-EVs show low CNS exposure after systemic delivery in primates, and the biological significance of this exposure remains to be established in neurodegenerative contexts. Second, administration route strongly determines systemic clearance and relative CNS access, with IT delivery providing the most reliable CNS exposure but limited by invasiveness, while IN delivery shows limited scalability to primates. Third, engineering strategies provide proof-of-principle that targeting can be shifted^[9]^. Rabies virus glycoprotein (RVG)-modified MSC-EVs improve neuronal uptake^[31]^, and ligand-guided approaches redirect EV tropism toward injured brain regions in rodent models^[37]^. Additionally, effective IN delivery in humans will likely require strategies that overcome mucociliary clearance and epithelial barriers. Approaches such as transient mucolytic pretreatment or surface modification remain insufficiently evaluated for MSC-EVs *in vivo*^[105,106]^. From a translational perspective, the central objective is therefore not simply to maximise brain uptake, but to define which level of exposure is sufficient for module regulation and to establish quantifiable robust biomarkers of CNS target engagement capable of anchoring dose selection and delivery strategy.

## MSC-EVS AS CANDIDATE MODULATORS OF POST-VIRAL NEURODEGENERATIVE RISK

Viral infections are increasingly recognised as environmental stressors capable of perturbing CNS homeostasis and potentially initiating or accelerating neurodegenerative processes. Epidemiological studies report associations between prior viral exposure and subsequent diagnosis of neurodegenerative disease, including elevated PD risk after hepatitis B or C, and the strong link between Epstein-Barr virus (EBV) and MS^[107-109]^. SARS-CoV-2 offers a contemporary framework for examining virus-associated neurodegenerative risk. Beyond acute infection, a substantial fraction of individuals develops neurological post-acute sequelae of SARS-CoV-2 infection (neuro-PASC), characterised by persistent cognitive impairment, fatigue, and sleep disturbances. Epidemiological analyses further report increased rates of dementia and parkinsonism within months after SARS-CoV-2 infection, although these observational associations do not establish causality^[110-113]^. Beyond these associations, experimental and post-mortem analyses indicate sustained microglial activation, vascular perturbation, mitochondrial stress, and early synaptic pathology after SARS-CoV-2 infection^[114-116]^. Moreover, biomarker profiling of long COVID cohorts identifies signatures consistent with neuronal injury, glial activation, and vascular disruption^[117-119]^. Recent conceptual frameworks propose that post-viral perturbations in the CNS, exemplified by SARS-CoV-2 infection, converge on the same inflammatory, mitochondrial, and proteostatic vulnerability modules described in the current review^[120]^. Evidence from other viral infections, such as EBV and herpes simplex virus, supports this convergent model^[121,122]^.

Considering their beneficial effects in neurodegenerative models, MSC-EVs are plausible candidates for modulating virus-associated CNS dysregulation and a putative therapeutic option for neuro-PASC. Clinical studies in severe COVID-19 have shown acceptable safety and preliminary anti-inflammatory or immunomodulatory signals for BM-MSC-EVs and UC-MSC-EVs, but these trials focused on acute respiratory disease rather than post-acute CNS pathology^[123-125]^. Evidence in long COVID remains limited, with early observations using UC-MSC-EVs providing feasibility data but not establishing efficacy for neuro-PASC^[126]^. Thus, current clinical evidence supports the plausibility of MSC-EV-mediated immune modulation during virus-associated disease but does not determine whether MSC-EVs can modulate the CNS changes associated with neuro-PASC.

A relevant proof-of-principle comes from a viral CNS disease model. In a progressive MS-like model induced by Theiler’s murine encephalomyelitis virus, intravenous administration of human AT-MSC-EVs attenuated glial reactivity, reduced brain atrophy, increased myelin protein expression, and improved motor function^[127]^. Although this model does not recapitulate SARS-CoV-2 infection, it demonstrates that MSC-EVs can modify virus-driven neuroinflammatory injury with neurodegenerative and demyelinating features *in vivo.* This supports evaluation of MSC-EVs in post-viral CNS dysregulation, while underscoring the need for direct SARS-CoV-2-relevant studies.

The three vulnerability modules outlined in the previous section provide a rationale for testing MSC-EVs in post-viral CNS dysregulation. In neuro-PASC, sustained glial activation and vascular perturbation can occur even in the absence of detectable viral replication^[128]^. NF-κB-dependent transcription and NLRP3 inflammasome activation have been implicated both in SARS-CoV-2-associated inflammation and in classical neurodegenerative processes^[129,130]^. MSC-EV-associated miRNAs such as miR-146a and miR-124 may intersect with these pathways by modulating NF-κB signalling and inflammasome activation, while altered circulating miRNA profiles in long COVID cohorts further support convergence at these regulatory nodes^[131,132]^. Long COVID cohorts exhibit evidence of oxidative DNA damage, glutathione depletion, mitochondrial fragmentation, impaired oxidative phosphorylation, and hypoxia-inducible factor (HIF)-1α signalling-driven inflammatory metabolic reprogramming^[133,134]^. Based on the effects observed in neurodegeneration models, MSC-EVs could be tested for their capacity to support mitochondrial biogenesis, mitophagy, restore oxidative phosphorylation capacity, and activate NRF2-dependent antioxidant responses in this setting [Figures 2 and 4]. In parallel, neuron-derived plasma EVs from individuals with neuro-PASC contain detectable nucleocapsid protein^[135]^. SARS-CoV-2 nucleocapsid protein has been linked to α-synuclein aggregation, neuronal toxicity *in vitro* and tau hyperphosphorylation through GSK3β activation *in vitro* and in mouse brain^[136]^, suggesting that viral protein persistence may contribute to early proteostatic stress. These findings provide a rationale to test whether MSC-EVs can enhance autophagy, modulate aggregation-related kinase signalling, and provide neurotrophic support in virus-stressed neural systems [Figure 4]. In addition, SARS-CoV-2 infection has been associated with demyelination and white-matter injury^[137,138]^, processes that MSC-EVs may counteract through remyelination, as shown in neurodegenerative models [Figure 4]. Direct evidence for these effects in SARS-CoV-2-associated neurodegeneration is still lacking.

**Figure 4:**
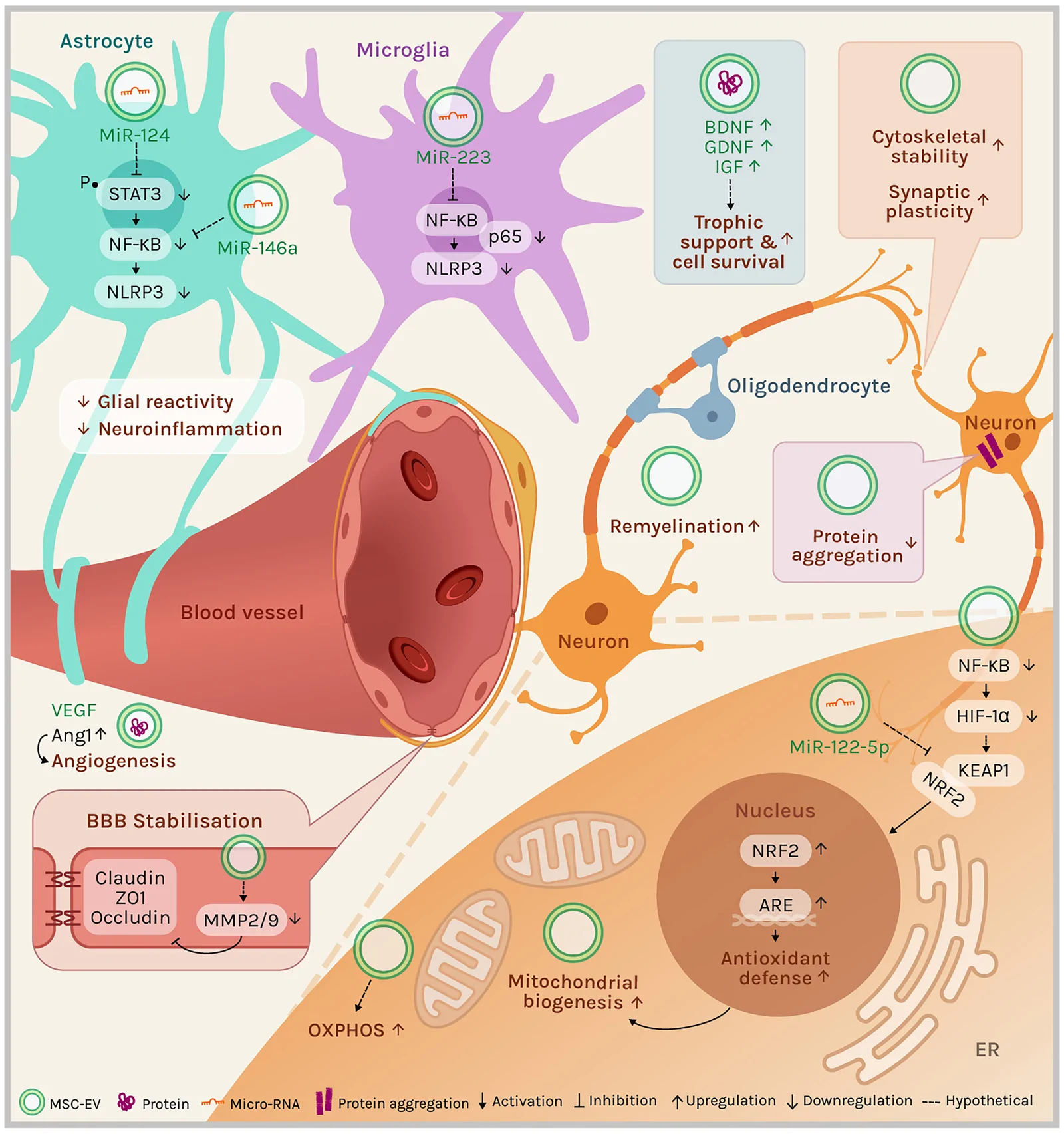
Conceptual framework illustrating how MSC-EVs may intercept convergent signalling pathways associated with post-viral infection neurodegenerative risk. Viral infections such as SARS-CoV-2 can generate a prolonged CNS stress state characterised by neuroinflammation with BBB dysfunction, mitochondrial and redox imbalance, and impaired proteostasis with declining trophic support. These interrelated vulnerability nodes may predispose virus-exposed neural tissue to neurodegenerative response. MSC-EV: Mesenchymal stem cell-derived extracellular vesicle; CNS: central nervous system; BBB: blood-brain barrier; SARS-CoV-2: severe acute respiratory syndrome coronavirus 2; NF-κB: nuclear factor kappa-light-chain-enhancer of activated B cells; STAT3: signal transducer and activator of transcription 3; NLRP3: NOD-like receptor family pyrin domain containing 3; BDNF: brain-derived neurotrophic factor; GDNF: glial cell line-derived neurotrophic factor; IGF-1: insulin-like growth factor 1; VEGF: vascular endothelial growth factor; Ang-1: angiopoietin-1; MMP-2/9: matrix metalloproteinase 2/9; ZO-1: zonula occludens-1; HIF-1α: hypoxia-inducible factor 1 alpha; KEAP1: Kelch-like ECH-associated protein 1; NRF2: nuclear factor erythroid 2-related factor 2; ARE: antioxidant response element; OXPHOS: oxidative phosphorylation; ER: endoplasmic reticulum.

Taken together, MSC-EVs may represent a potential therapeutic option for virus-associated neurodegeneration, analogous to their beneficial effects in classical models. However, their relevance to post-viral CNS disease remains hypothetical. Establishing the relevance of MSC-EV-based treatment will require infection-relevant models that capture disease stage, CNS target engagement, and functional outcomes, including virus-exposed human brain organoids^[139]^, neurovascular models, and *in vivo* paradigms combining viral stress with genetic susceptibility^[140]^. Such approaches will be essential to determine whether MSC-EVs can engage these convergent signalling modules in post-viral CNS models and whether such engagement translates into measurable functional benefit.

MSC-EV cargo may intersect with these regulatory networks through several mechanisms. EV-miRNAs (e.g., miR-146a, miR-124) may dampen NF-κB and inflammasome-dependent inflammatory signalling in glial cells, potentially restoring inflammatory homeostasis and BBB stability. MSC-EVs may support mitochondrial quality control by enhancing mitophagy, promoting mitochondrial biogenesis, and activating NRF2-dependent antioxidant responses, thereby improving cellular redox balance. MSC-EVs may also reinforce neuronal resilience by enhancing clearance of aggregation-prone proteins and delivering neurotrophic factors such as BDNF and GDNF. MSC-EV signalling may further support remyelination during virus-associated white matter injury.

The interactions depicted represent a hypothetical framework derived from MSC-EV activity observed in other neurodegenerative and inflammatory settings. Direct evidence demonstrating MSC-EV modulation of these pathways in SARS-CoV-2-associated neurological disease remains limited and requires validation in infection-relevant experimental systems.

## DISCUSSION

Neurodegenerative diseases are commonly defined by their dominant proteinopathies or regional vulnerabilities. However, accumulating evidence indicates that disease progression reflects convergence at a limited set of regulatory nodes that govern inflammatory tone, mitochondrial resilience, and proteostatic balance^[6]^. The framework synthesised in this review organises diverse observations of MSC-EV activity into a coherent mechanistic architecture aligned with these convergent stress-integration modules. By situating MSC-EV-mediated effects at the level of these shared modules, this perspective delineates how MSC-EVs may engage interconnected regulatory circuits that couple inflammatory thresholds, redox control, and intracellular protein quality management to neuronal survival and network stability [Figures 1-3]. Framing disease progression in terms of these interacting modules shifts emphasis away from cataloguing individual dysregulated molecules and toward identifying regulatory points that influence disease trajectory. At the same time, this framework should be interpreted with appropriate caution, as many studies rely on EV-enriched preparations in which co-isolated proteins, lipoproteins, or other soluble factors may contribute to the observed biological effects.

This modular perspective helps address several recurring ambiguities in the MSC-EV field. First, it explains how MSC-EVs can produce coordinated effects across seemingly disparate pathological readouts without being mechanistically indiscriminate. MSC-EV treatment has been associated with modulation of defined signalling axes, including NF-κB-linked inflammatory regulation, NRF2-associated antioxidant programs, and autophagy-lysosomal pathways, which in turn orchestrate multiple downstream outputs^[30,33,56]^. Second, it explains how limited EV penetration may nevertheless produce system-level biological effects. Modulation at neurovascular and glial interfaces may reshape network-level stress responses without requiring extensive neuronal uptake, allowing relatively modest EV penetration to generate amplified biological consequences^[96,97]^. Together, these observations suggest that the apparent pleiotropy of MSC-EVs reflects engagement at regulatory nodes that coordinate inflammatory, metabolic, and proteostatic stability.

Translating this mechanistic framework into therapeutic benefit will require that MSC-EVs engage these regulatory modules with sufficient consistency, exposure, and reproducibility *in vivo*. Despite growing mechanistic insight into MSC-EV activity, therapeutic variability across studies likely reflects differences in EV composition, purity, potency, dosing, and delivery efficiency. MSC tissue source, culture conditions, and isolation strategies influence EV cargo and co-isolated factors, including proteins, lipoproteins, and soluble mediators, making it difficult in some studies to attribute biological effects exclusively to EV-associated cargo^[87,141]^. This remains a central limitation in the field and underscores the need for experimental designs that distinguish EV-mediated activity from the effects of non-vesicular components. Moreover, MSC-EV preparations are heterogeneous, comprising EV subpopulations that may differ in cargo composition, tropism, uptake route, and functional activity. Bulk particle measurements therefore provide limited information about which EV subsets reach relevant target cells or drive specific biological outcomes^[142]^. Systemic administration further leads to rapid peripheral sequestration and limited, context-dependent CNS exposure, complicating efforts to define dose-engagement-response relationships. Beyond standardisation, engineering and priming strategies may further improve therapeutic performance by enhancing CNS targeting and biasing MSC-EV cargo toward module-specific mechanisms of action, as outlined in [Box 1]. Interpretation of biodistribution studies is further complicated by differences in EV labelling and tracking strategies, which may not always distinguish intact vesicle delivery from label transfer, degradation products, or uptake of non-vesicular material^[39,104]^. Progress toward clinical application will therefore require more rigorous standardisation of MSC-EV production pipelines, including Good Manufacturing Practice (GMP)-compatible manufacturing, orthogonal characterisation of EV preparations, validated potency assays linked to mechanistic module engagement, and reproducible dose metrics that extend beyond particle number alone^[12]^. Although MSC-EVs have generally demonstrated a favourable safety profile, the consequences of repeated administration and systemic immune modulation remain incompletely understood. Addressing these issues will require carefully designed studies integrating biodistribution analysis, pharmacodynamic biomarkers of CNS target engagement, and longitudinal monitoring of molecular and functional outcomes.

The modular framework outlined here may also have implications beyond established neurodegenerative disease. Viral infections such as SARS-CoV-2 can induce sustained neurovascular activation, mitochondrial stress, and impaired proteostasis that persist beyond the acute phase yet precede overt neuronal loss^[120]^. Conceptualising these changes within the same stress-regulatory modules suggests that virus-associated neurological syndromes may represent early states of network instability rather than fully autonomous neurodegenerative pathology. In this context, MSC-EVs may serve not only as therapeutic candidates but also as experimental probes capable of testing whether destabilised inflammatory, metabolic, and proteostatic circuits remain reversible during this intermediate phase.

Taken together, the synthesis presented in this review positions MSC-EVs as module-directed regulators of stress-integration networks. This perspective provides a stronger mechanistic basis for evaluating MSC-EVs across neurodegenerative diseases and related conditions characterised by convergent inflammatory, metabolic, and proteostatic dysregulation. Emerging early-phase data, including pilot observations in ALS demonstrating safety and signals of functional stabilisation following repeated BM-MSC-EV administrations^[143]^, provide preliminary clinical alignment with this conceptual framework. Realising the translational potential of MSC-EVs will therefore require clinical strategies that directly test whether these EVs engage the regulatory modules identified here in patients. This will depend on integrating quantitative measures of EV exposure with pharmacodynamic biomarkers that report modulation of inflammatory, metabolic, and proteostatic pathways *in vivo*. Emerging molecular stratification approaches further support this direction. For example, recent CSF proteomic studies have identified biologically distinct AD subtypes characterised by inflammatory, metabolic, and synaptic signatures, highlighting the heterogeneity of underlying disease mechanisms and the potential need for module-directed therapeutic strategies^[144]^. In this context, MSC-EVs may ultimately inform precision therapeutic strategies targeting the stress-regulatory modules driving disease in individual patients.

## CONCLUSION

MSC-EVs hold therapeutic promise in neurodegenerative diseases because they engage interconnected regulatory pathways rather than acting through a single cargo molecule or disease-specific mechanism. The evidence reviewed here supports a model in which MSC-EVs modulate three convergent stress-integration modules: neuroinflammation and BBB dysfunction, mitochondrial and redox imbalance, and impaired proteostasis with declining neurotrophic support. This modular view helps explain their pleiotropic effects across diverse neurodegenerative contexts and provides a rationale for more mechanism-guided therapeutic development. However, clinical translation will depend on moving beyond descriptive efficacy toward demonstrable target engagement. Future studies should define how MSC-EV source, cargo composition, delivery route, biodistribution, and dosing influence engagement of disease-relevant CNS pathways. Integrating pharmacodynamic biomarkers with functional outcomes will be essential to determine whether MSC-EVs can be developed as reproducible, module-directed interventions for neurodegenerative diseases.

## DECLARATIONS

### AI and AI-assisted tools statement

During the preparation of this manuscript, the AI tool ChatGPT (version GPT5.5 Thinking, released 2026-04-23) was used solely for language editing and structural refinement to improve clarity and readability. The tool did not influence the study design, data collection, analysis, interpretation, or the scientific content of the work. All authors take full responsibility for the accuracy, integrity, and final content of the manuscript.

### Copyright

© The Author(s) 2026.
